# Relative Age in School and Initiation of Speech Therapy in Children

**DOI:** 10.1001/jamanetworkopen.2025.12262

**Published:** 2025-05-23

**Authors:** Sophie Billioti de Gage, Hugo Peyre, Martin Chalumeau, Yann Mikaeloff, Mahmoud Zureik, Alain Weill

**Affiliations:** 1 EPI-PHARE, Epidemiology of Health Products (French National Agency for the Safety of Medicines and Health Products and French National Health Insurance), Saint-Denis, France; 2 Centre de Ressources Autisme Languedoc-Roussillon et Centre d’Excellence sur l’Autisme et les Troubles Neuro-développementaux (CeAND), CHU Montpellier, Montpellier, France; 3 Laboratoire de Sciences Cognitives et Psycholinguistique, Département d’Études Cognitives, Ecole Normale Supérieure, PSL University, EHESS, CNRS, Paris, France; 4 Obstetrical, Perinatal and Pediatric and Life Course Epidemiology Research Team, Centre for Research in Epidemiology and Statistics, Université Paris Cité, Inserm, Paris, France; 5 Service de pédiatrie générale et maladies infectieuses, Hôpital Necker-Enfants malades, AP-HP, Université Paris Cité, Paris, France; 6 Centre de protection de l’enfant et de l’adolescent, Hôpital Paul-Brousse AP-HP, Villejuif, France; 7 Centre for Epidemiology and Population Health, Inserm, Paris-Saclay, Villejuif, France; 8 Université Versailles Saint-Quentin-en-Yvelines, Université Paris Saclay, Guyancourt, France

## Abstract

**Importance:**

In most countries, children in the same grade can be up to 12 months apart in age, which represents a considerable relative age difference, particularly in the early years. A relative age effect has been frequently reported in the diagnosis and treatment of attention-deficit/hyperactivity disorder and, more rarely, in the diagnosis of other neurodevelopmental disorders. The relative age effect has never been studied for speech therapy, which is frequently prescribed to treat specific language and learning disorders.

**Objective:**

To quantify the association of relative age with the initiation of speech therapy.

**Design, Setting, and Participants:**

This cohort study used data from the French National Health Data System and included all children born in France between 2010 and 2016 from September of the year of their 5th birthday until July of the year of their 10th birthday or July 31, 2022 (end of study).

**Exposure:**

Children were categorized according to month of birth.

**Main Outcomes and Measures:**

Risk of speech therapy initiation was estimated using Cox models adjusted for other known risk factors.

**Results:**

In total, 4 188 985 children (mean [SD] age, 5.2 [0.3] years; 50.8% girls) were included. Speech therapy was initiated for 692 086 children (incidence rate, 53.1 per 1000 person-years, 54.5% boys) during a mean (SD) follow-up of 3.1 (1.6) years. Among children of the same schooling level, the risk of initiating speech therapy steadily increased according to quarter and month of birth, with adjusted hazard ratios of 1.51 (95% CI, 1.50-1.52) for children born in the last quarter vs the first quarter and 1.64 (95% CI, 1.62-1.66) for children born in December vs those born in January. The relative age effect observed for speech therapy was of the same magnitude as that observed for methylphenidate in attention-deficit/hyperactivity disorder (positive control outcome). No relative age effect was observed for the initiation of desmopressin for nocturnal enuresis (negative control outcome).

**Conclusions and Relevance:**

This cohort study found that among children in the same grade, an age difference of a few months was associated with the frequency of initiation of speech therapy. These findings may be attributable to a mismatch between the expectations of the school system and the relative age and level of maturity of the children or earlier identification of neurodevelopmental disorders in the youngest children in each age group.

## Key Points

**Question:**

What is the association between relative age and initiation of speech therapy in children aged 5 to 10 years?

**Findings:**

In this cohort study of 4 188 985 school-aged children, within the same grade, those born in December (the youngest) were 64% more likely to start speech therapy than those born in January (the oldest). The increase in risk was constant for each month of birth from February to December.

**Meaning:**

These findings suggest that age-related relative immaturity may be misdiagnosed as a learning disability or relative maturity may lead to underdiagnosis of this disorder and that professionals in the field should adapt their practices accordingly.

## Teaser

This cohort study used French national health system data to evaluate the association of relative age with the initiation of speech therapy among school-aged children in the same the grade.

## Introduction

Specific language and learning disorders are among the most prevalent neurodevelopmental disorders,^1,2^ affecting approximately 8%^3,4^ of school-aged children. These disorders can affect 1 or more cognitive functions, including oral language (difficulties in language comprehension and expression, articulation, fluency, and voice quality),^5^ academic learning (reading, written expression, mathematics),^6^ and gestural and/or visuospatial and transversal functions (attention, memory, executive functions),^7^ and account for a substantial proportion of educational difficulties and failed academic performance.^6,8^ In France, speech therapy, mainly delivered in private practice (ie, outside a hospital or school),^9^ is often prescribed to treat these disorders.^7,10,11^ Studies conducted in France estimated that nearly 10% of children and adolescents younger than 19 received speech therapy in 2019^10^ and that the use of speech therapy for children aged 6 years or older was most often initiated by teachers or parents.^7,9^ Several pre-, peri-, and postnatal factors have been associated with the likelihood of prescribing speech therapy, including male sex, prematurity, and socioeconomic factors.^12,13^

The relative age effect is the tendency, within age cohorts, of individuals born earlier (ie, older) to perform better. It has been observed in several contexts, notably in top-level sports and academic achievement.^14,15^ Indeed, children in the same grade can be up to 12 months apart in age, representing a substantial relative difference in physical development and cognitive maturity, particularly in the early years. Several international studies have shown that younger children are more likely to be diagnosed or treated for attention-deficit/hyperactivity disorder (ADHD) than older children in the same grade.^16^ Fewer studies have shown the same association with the diagnoses of other neurodevelopmental disorders,^17^ such as autism,^18,19^ intellectual development,^19,20^ developmental motor coordination,^21^ and learning disorders.^22^ The relative age effect has never been studied for speech therapy prescriptions, which may be associated with neurologic maturation and may be influenced by this effect.

We aimed to quantify the relative age effect on speech therapy initiation among children aged 5 to 10 years in France and to identify potential modulating factors. The magnitude of the effect was compared with that assessed for a positive control outcome (ie, initiation of methylphenidate for ADHD). A negative control outcome (ie, initiation of desmopressin for nocturnal enuresis) was also studied to test the influence of school environment.

## Methods

### Design and Setting

This cohort study was conducted using the French National Mother-Child (known as EPI-MERES) register,^23^ which was developed from the French National Health Data System on the basis of algorithms published in previous studies.^24,25,26^ This study was granted permanent regulatory access to EPI-PHARE. No specific authorization or need for informed consent from the French data protection authority was required. The study followed to the Strengthening the Reporting of Observational Studies in Epidemiology (STROBE) reporting guideline.^27^

The EPI-MERES register includes all pregnancies managed in France since January 2010. For pregnancies resulting in delivery, the mother’s and child’s information is linked using a unique identifier. The French National Health Data System contains sociodemographic and medical data on all outpatient services reimbursed by national health insurance since 2006, including prescribed drugs and expenditures for long-term diseases and medical and paramedical visits. It also includes diagnoses linked to hospital admissions and procedures performed during hospital stays.^28,29^

### Study Population and Follow-Up

Children born between 2010 and 2016 were included on September 1 of the year of their 5th birthday from 2015 to 2021. Children with congenital malformations or chromosomal abnormalities (eg, Down syndrome); mental, behavioral, or developmental disorders (eg, stress-related disorders, intellectual disability, speech and language disorders, autism spectrum disorders); nervous system disorders (eg, epilepsy); or psychotropic drug prescriptions (ie, anxiolytics, hypnotics, antidepressants, psychostimulants, mood regulators) before study entry were excluded (eTable 1 in Supplement 1). Children who had received speech therapy before inclusion were also excluded. Children were followed up from inclusion date until the first of the following events: speech therapy initiation, loss to follow-up, death, July 31 of the year of their 10th birthday, or the end of the study period (July 31, 2022) (eFigure 1 in Supplement 1).

### Relative Age

Date of birth (defined either by quarter or month) was considered as a discrete variable used to estimate age differences within a given grade. In France, children start school the year they turn 3 years, and the school year runs from September to early July. The birth deadline for entering the school year is December 31 (eTable 2 in Supplement 1). Thus, children born in January are generally the oldest and those born in December are the youngest in their class. In France, repeating a school year has been practiced only exceptionally for children younger than 10 years since 2015, and grade skipping is rare. Furthermore, there is no provision in France for delaying school entry for children born at the end of the year (ie, generally the youngest), meaning that age differences within the same class can be approximated by the month of birth.

### Outcomes and Covariates

The main outcome was speech therapy initiation defined as the first reimbursement since inclusion. Sociodemographic data included sex and at childbirth, the mother’s affiliation with solidarity-based complementary health insurance (free access to health care for people with a low income), disadvantage index of the municipality of residence (quintiles, the first corresponding to the most advantaged level),^30^ size of urban area by number of inhabitants, and region of residence. Pregnancy and delivery characteristics were gestational age; birth weight adjusted for age and sex *z* score^31^; birth rank among siblings (assessed using information on hospital stays at birth that have been available since 2006); and in utero exposure to tobacco (indicator), alcohol (indicator), psychotropic drugs, and valproic acid. The calendar year of inclusion was also considered.

### Statistical Analysis

#### Descriptive Analyses

Incidence rates of speech therapy use were calculated (1) overall and (2) according to birth month and inclusion year for the 3 cohorts of children with a maximum observation period (ie, children included between 2015 and 2017 followed at most until July 31 of the year of their 10th birthday). The characteristics of the children initiating speech therapy are described in the Results.

#### Main Analyses

Survival analyses using Cox models were performed to estimate the association between date of birth and speech therapy initiation. The explanatory variable of interest was either the quarter or month of birth. The reference values considered for the quarter and month of birth were (1) the first quarter and the month of January to measure the widest amplitude of a potential relative age effect or (2) the second quarter and the month of June to approximate the median maturity within an age group. The models were adjusted for the aforementioned covariates.

#### Subgroup Analyses

Subgroup analyses were conducted to assess potential modulating factors of the relative age effect on speech therapy initiation according to sex, affiliation with solidarity-based complementary health insurance, disadvantage index of the municipality of residence, birth rank among siblings, and prematurity at birth. The birth date considered was the half-year, with the first half of the year as the reference.

#### Sensitivity Analyses

Several sensitivity analyses were conducted. First, we assessed whether the relative age effect was influenced by the school grade by varying the follow-up time, interrupting it successively on July 31 of the year in which the child turned (1) 6 years (end of nursery school), (2) 7 years (end of first elementary class), (3) 8 years (end of second elementary class), and (4) 9 years (end of third elementary class), with all children being followed up until July 31 of the year of their 10th birthday (end of fourth elementary class) in the main analysis (eTable 2 in Supplement 1). Second, analyses were stratified according to the calendar year of inclusion to assess the period effect, as the use of speech therapy may have changed over time. Third, we assessed whether the relative age effect differed between children who received therapy or not after an initial assessment by the speech therapist. Indeed, the initial assessment should lead to a reduction in a potential relative age effect by considering the child’s actual age.

#### Analyses With Positive and Negative Control Outcomes

Descriptive and main analyses were replicated on a positive control outcome (ie, initiation of methylphenidate for ADHD [Anatomical Therapeutic Chemical code N06BA04]) to validate our results and compare the magnitude of potential associations between date of birth and speech therapy initiation. The relative age effect was also assessed for desmopressin initiation (mainly indicated for nocturnal enuresis, the diagnosis or management of which does not appear to be predominantly influenced by the school environment [Anatomical Therapeutic Chemical code H01BA02]), which was considered as a negative control outcome to test the influence of the school environment on the main results.

The data were analyzed using SAS Enterprise Guide, version 8.3 (SAS Institute Inc). A 95% confidence interval around the hazard ratios that did not include 1 defined statistical significance.

## Results

### Population and Outcomes

**Figure 1.**
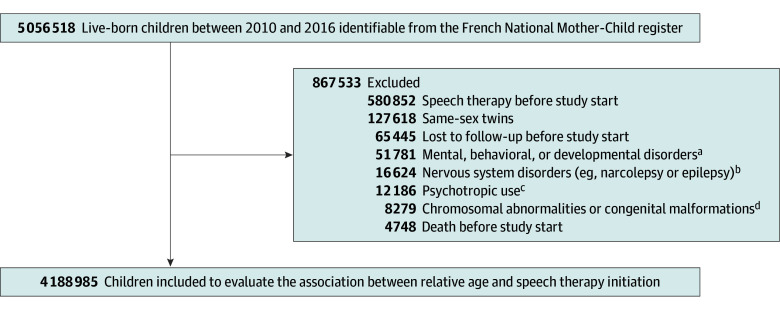
Study Flow Diagram ^a^Including intellectual disability (n = 8705); pervasive developmental disorders (n = 22 420); other disorders of psychological development (n = 10 712); hyperkinetic disorders (n = 768); other behavioral and emotional disorders with onset usually occurring in childhood and adolescence (5667); neurotic, stress-related, or mood disorders (n = 1249); and schizophrenic, schizotypal, and delusional disorders (n = 80). ^b^Including epilepsy (n = 16 614) and narcolepsy (n = 10). ^c^Including anxiolytics, hypnotics, and sedatives (n = 10 699); antipsychotics (n = 938); antidepressants or mood regulators (n = 317); and methylphenidate (n = 232). ^d^Including Down syndrome (n = 3240).

**Figure 2.**
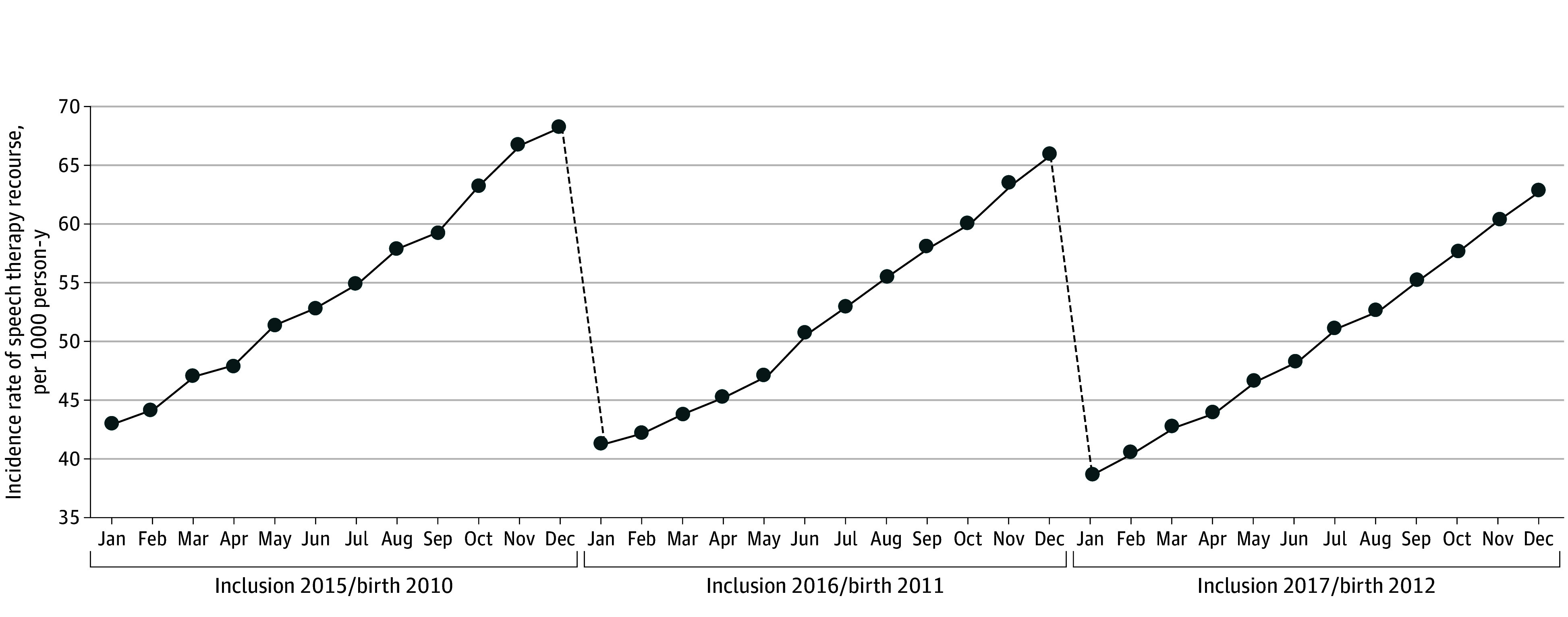
Incidence of Recourse to Speech Therapy by Birth Month and Year of Study Entry for Children Born Between 2010 and 2012 Who Received the Maximum Follow-Up Dashed lines illustrate the sharp decrease in incidence of recourse to speech therapy for children born at the beginning of a year vs those born at the end of the previous year.

In total, 4 188 985 children (mean [SD] age, 5.2 [0.3] years; 49.2% boys and 50.8% girls) were included (Figure 1). A first speech therapy session was initiated for 692 086 children (incidence rate, 53.1 per 1000 person-years, 54.5% boys and 45.5% girls) during a mean (SD) follow-up of 3.1 (1.6) years. The characteristics of speech therapy initiators are described in the Table. The mean (SD) age at initiation was 6.9 (1.3) years. Compared with children without recourse to speech therapy during follow-up, those with a first recourse were more likely to be boys (54.5% vs 48.1%); to be exposed in utero to tobacco (8.4% vs 7.0%), alcohol (0.6% vs 0.5%), or psychotropic drugs (4.7% vs 3.7%); to be born prematurely (6.5% vs 5.6%); to be born with a low weight for gestational age (12.0% vs 11.0%); to ranked as the second sibling (35.9% vs 34.3%); to live in a more privileged municipality (41.2% vs 38.2% for the first 2 quintiles of the disadvantage index); or to live in an urban area of fewer than 10 000 inhabitants (35.9% vs 32.3%). There were no marked differences between children with a first speech therapy session and those without in terms of affiliation to the solidarity-based complementary health insurance or in utero exposure to valproic acid. Compared with children who did not receive speech therapy, children receiving it were more often born in the last quarter of the year (29.9% vs 24.1%); more likely to live in the Auvergne-Rhône-Alpes (13.7% vs 11.6%), Hauts de France (10.6% vs 8.6%), or Occitanie (10.2% vs 8.0%) regions; and less likely to live in the Ile-de-France (including Paris) (13.6% vs 21.4%) or French overseas (2.3% vs 4.1%) regions. For each of the 3 cohorts of children with a maximal observation period, there was a relatively regular increase in the incidence of speech therapy use according to the month of birth from January to December and a systematic sharp decrease in incidence for children born at the beginning of a year vs those born at the end of the previous year (December 2010 to January 2011, from 68.3 to 41.3 per 1000 person-years; December 2011 to January 2012, from 65.8 to 38.7 per 1000 person-years) (Figure 2).

**Table.** Characteristics of Children Initiating Speech Therapy Between the Years of Their 5th and 10th Birthdays

Characteristic	Children, No. (%)	
	Treated with speech therapy (n = 692 086)	Not treated with speech therapy (n = 3 496 899)
Sex		
Female	314 813 (45.5)	1 813 865 (51.9)
Male	377 273 (54.5)	1 683 034 (48.1)
Gestational age, wk		
Nonmissing	692 073 (100.0)	3 496 819 (100.0)
22-27 (Extremely preterm)	1374 (0.2)	4171 (0.1)
28-31 (Very preterm)	4227 (0.6)	15 956 (0.5)
32-36 (Moderate to late preterm)	39 232 (5.7)	174 661 (5.0)
37-40 (Full term)	523 871 (75.7)	2 667 410 (76.3)
>40 (Term exceeded or post term)	123 369 (17.8)	634 621 (18.1)
Weight for gestational age, percentile^a^		
Nonmissing	663 215 (95.8)	3 380 500 (96.7)
<3rd (Severe low weight)	29 870 (4.3)	129 868 (3.7)
<10th (Small for gestational age)	53 315 (7.7)	255 559 (7.3)
10-90th (Normal weight)	506 201 (73.1)	2 615 275 (74.8)
>90th (Macrosomia)	43 926 (6.3)	226 888 (6.5)
>97th (Severe macrosomia)	29 903 (4.3)	152 910 (4.4)
In utero exposure vs nonexposure		
Tobacco	58 177 (8.4)	244 901 (7.0)
Alcohol	4483 (0.6)	18 828 (0.5)
Psychotropic drugs	32 403 (4.7)	129 496 (3.7)
Valproic acid	761 (0.1)	2761 (0.1)
Birth rank among siblings^b^		
Nonmissing	691 840 (100.0)	3 495 886 (100.0)
1	401 343 (58.0)	2 042 807 (58.4)
2	248 523 (35.9)	1 198 037 (34.3)
≥3	41 974 (6.1)	255 042 (7.3)
Solidarity-based complementary health insurance		
Nonmissing	691 011 (99.8)	3 491 012 (99.8)
No	621 649 (89.8)	3 119 832 (89.2)
Yes	69 362 (10.0)	371 180 (10.6)
Disadvantage index, quintile^c^		
Nonmissing	663 787 (95.9)	3 292 826 (94.2)
1 (Most advantaged)	138 525 (20.0)	670 561 (19.2)
2	146 602 (21.2)	665 070 (19.0)
3	136 533 (19.7)	646 466 (18.5)
4	126 418 (18.3)	646 876 (18.5)
5 (Least advantaged)	115 709 (16.7)	663 853 (19.0)
Size of urban area of residence, No. of inhabitants		
Nonmissing	677 389 (97.9)	3 421 104 (97.8)
<2000	151 960 (22.0)	699 963 (20.0)
2000-9999	95 998 (13.9)	431 280 (12.3)
10 000-49 999	70 233 (10.1)	379 604 (10.9)
50 000-199 999	81 526 (11.8)	450 263 (12.9)
≥200 000	277 672 (40.1)	1 459 994 (41.8)
French region of residence		
Nonmissing	692 084 (100.0)	3 496 865 (100.0)
Auvergne-Rhône-Alpes	94 967 (13.7)	406 718 (11.6)
Bourgogne-Franche-Comté	24 935 (3.6)	134 983 (3.9)
Bretagne	40 694 (5.9)	161 749 (4.6)
Centre-Val de Loire	24 071 (3.5)	139 518 (4.0)
Corse	2878 (0.4)	11 479 (0.3)
Grand Est	58 333 (8.4)	244 227 (7.0)
Hauts-de-France	73 432 (10.6)	300 235 (8.6)
Ile-de-France	93 794 (13.6)	749 306 (21.4)
Normandie	31 135 (4.5)	176 557 (5.0)
Nouvelle-Aquitaine	56 786 (8.2)	289 542 (8.3)
Occitanie	70 724 (10.2)	279 352 (8.0)
Pays de la Loire	43 959 (6.4)	206 764 (5.9)
Provence-Alpes-Côte d’Azur	60 224 (8.7)	253 535 (7.3)
French overseas region	16 152 (2.3)	142 900 (4.1)
Year of 5th birthday^d^		
2015	134 324 (19.4)	444 330 (12.7)
2016	133 297 (19.3)	465 523 (13.3)
2017	131 183 (19.0)	479 250 (13.7)
2018	112 123 (16.2)	494 300 (14.1)
2019	88 732 (12.8)	520 829 (14.9)
2020	63 092 (9.1)	539 771 (15.4)
2021	29 335 (4.2)	552 896 (15.8)
Quarter of birth		
January-March	138 075 (20.0)	877 604 (25.1)
April-June	157 451 (22.8)	874 124 (25.0)
July-September	189 464 (27.4)	901 237 (25.8)
October-December	207 096 (29.9)	843 934 (24.1)
Month of birth		
January	46 198 (6.7)	305 620 (8.7)
February	43 491 (6.3)	278 331 (8.0)
March	48 386 (7.0)	293 653 (8.4)
April	48 582 (7.0)	282 661 (8.1)
May	53 707 (7.8)	299 349 (8.6)
June	55 162 (8.0)	292 114 (8.4)
July	61 672 (8.9)	308 223 (8.8)
August	63 457 (9.2)	300 175 (8.6)
September	64 335 (9.3)	292 839 (8.4)
October	68 249 (9.9)	294 265 (8.4)
November	67 278 (9.7)	272 635 (7.8)
December	71 569 (10.3)	277 034 (7.9)

^a^ Adjusted for gestational age and sex *z* score.

^b^ Assessed using information on hospital stays at birth since 2006.

^c^ Disadvantage index of the municipality of residence.

^d^ Due to the study design, the maximum duration of follow-up was shorter for children included after 2017.

### Main Analyses

**Figure 3.**
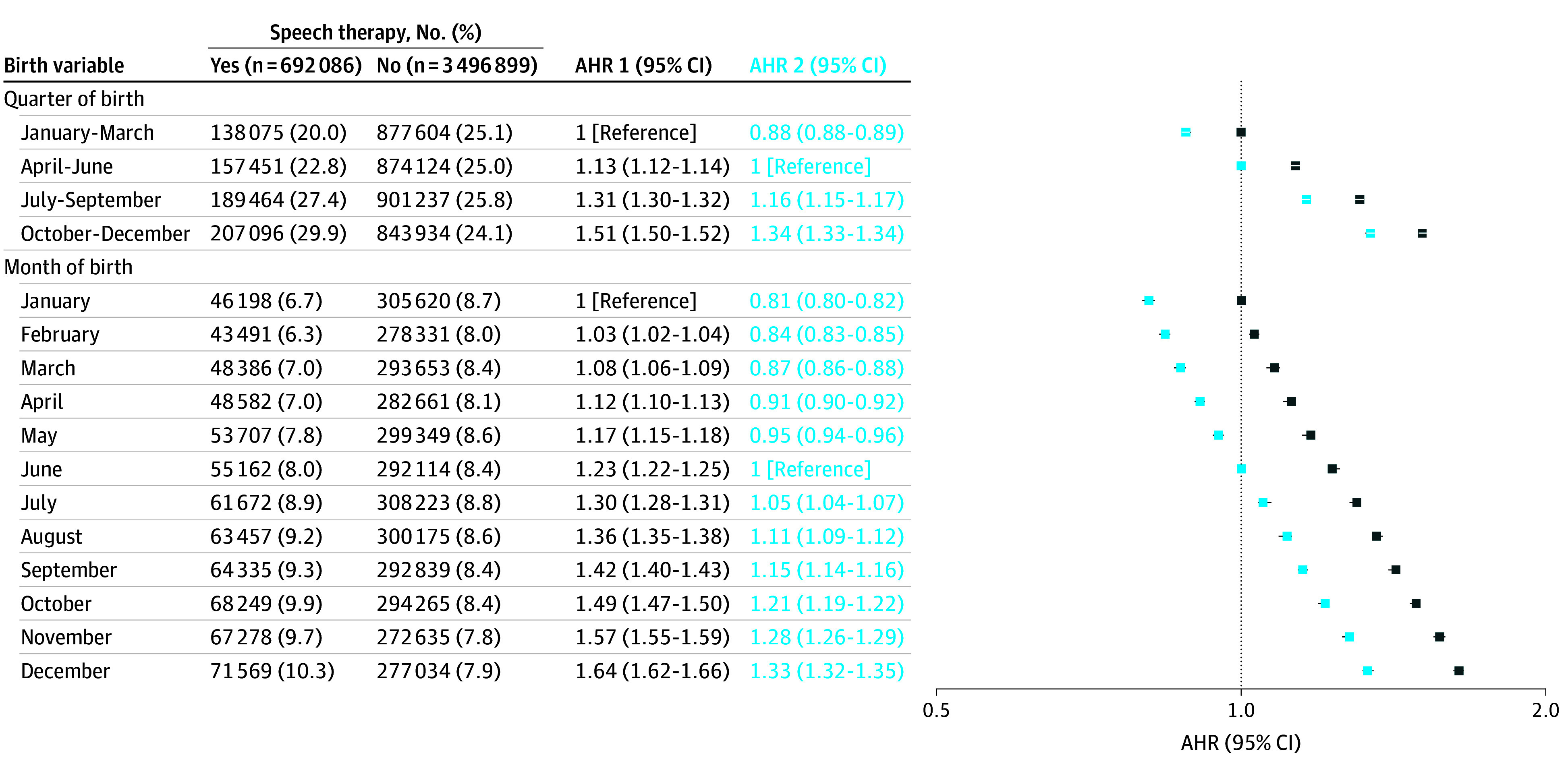
Associations Between the Date of Birth (Quarter or Month) and the Initiation of Speech Therapy Between the Years of the 5th and 10th Birthdays Models were adjusted for birth quarter (January-March as reference for adjusted hazard ratio 1 [AHR 1] and April-June as reference for AHR 2) or month (January as reference for AHR 1 and June as reference for AHR 2); sex; gestational age; weight for gestational age; birth rank among siblings; in utero exposure to tobacco, alcohol, psychotropic drugs, or valproic acid; solidarity-based complementary health insurance; disadvantage index; size of urban area of residence; French region of residence; and calendar year of inclusion.

After adjustment, we observed a steady increase in the risk of speech therapy initiation according to quarter and month of birth, with adjusted hazard ratios (AHRs) of 1.51 (95% CI, 1.50-1.52) for children born in the last quarter vs those born in the first quarter and 1.64 (95% CI, 1.62-1.66) for children born in December vs those born in January. Compared with children born in the second quarter, the risk of initiating speech therapy increased for children born in the last quarter (AHR, 1.34 [95% CI, 1.33-1.34]) and decreased for children born in the first quarter (AHR, 0.88 [95% CI, 0.88-0.89]). Compared with children born in June, there was a steady increase in the risk of speech therapy initiation for children born after June, with an AHR of 1.33 (95% CI, 1.32-1.35) for children born in December, and a decrease for children born before June, with an AHR of 0.81 (95% CI, 0.80-0.82) for children born in January (Figure 3).

**Figure 4.**
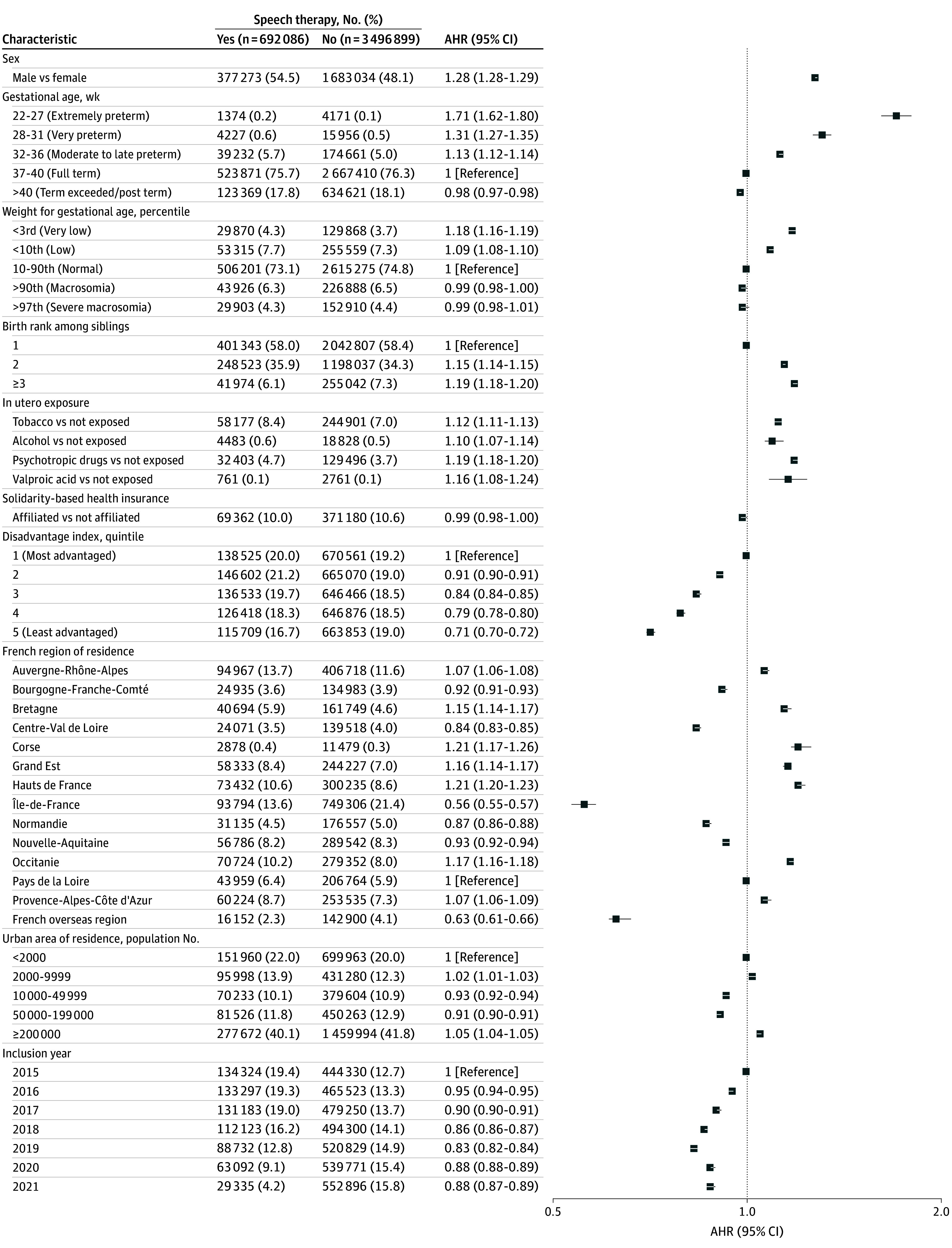
Association Between Adjustment Factors and the Initiation of Speech Therapy Models were adjusted for birth quarter; sex; gestational age; weight for gestational age; birth rank among siblings; in utero exposure to tobacco, alcohol, psychotropic drugs, or valproic acid; solidarity-based complementary health insurance; disadvantage index of municipality of residence; size of urban area of residence; French region of residence; and calendar year of inclusion. AHR indicates adjusted hazard ratio.

The other main factors significantly associated with speech therapy initiation were male sex (AHR, 1.28 [95% CI, 1.28-1.29]); prematurity (for extreme vs no prematurity: AHR, 1.71 [95% CI, 1.62-1.80]); low weight for gestational age (for very low vs normal weight: AHR, 1.18 [95% CI, 1.16-1.19]); birth rank among siblings (second child: AHR, 1.15 [95% CI, 1.14-1.15]; third child or more: 1.19 [95% CI, 1.18-1.20]); in utero exposure to tobacco (AHR, 1.12 [95% CI, 1.11-1.13]), alcohol (AHR, 1.10 [95% CI, 1.07-1.14]), psychotropic drugs (AHR, 1.19 [95% CI, 1.18-1.20]), or valproic acid (AHR, 1.16 [95% CI, 1.08-1.24]); and living in a more advantaged municipality (for the least vs the most advantaged quintile: AHR, 0.71 [95% CI, 0.70-0.72]). There were significant variations in the risk of initiating speech therapy according to region of residence, with a notably lower probability for the Ile-de-France (that includes Paris) region (AHR, 0.56 [95% CI, 0.55-0.57]) (Figure 4).

### Subgroup and Sensitivity Analyses

The association of date of birth with speech therapy initiation was broadly similar in all subgroup analyses (eTable 3 in Supplement 1). A relative age effect was observed regardless of grade, but was more marked between age 5 and 7 years (eTable 4 in Supplement 1). The relative age effect was similar regardless of the year of inclusion (eTable 5 in Supplement 1) and was slightly higher for children who received therapy than for those who only underwent a single assessment not followed by therapy (eTable 6 in Supplement 1).

### Analyses With Positive and Negative Control Outcomes

Methylphenidate was initiated for 38 794 children (incidence rate, 2.3 per 1000 person-years) (eTable 7 in Supplement 1). As observed for speech therapy, the incidence of methylphenidate prescriptions (1) increased fairly steadily with the month of birth (from January to December) for children in the same school year and (2) decreased sharply (from 30% to 37%) between children born at the end of the year and those born at the beginning of the following school year (eFigure 2 in Supplement 1). Adjusted Cox models indicated a steady increase according to quarter and month of birth, with an AHR of 1.55 (95% CI, 1.47-1.62) for children born in December vs January (eFigure 3 in Supplement 1). Desmopressin was initiated for 58 589 children (incidence rate, 3.6 per 1000 person-years) (eTable 7 in Supplement 1). No relative age effect was found for desmopressin initiation (eTable 8 in Supplement 1).

## Discussion

This cohort study performed in a nearly exhaustive population of children aged 5 to 10 years living in France shows that the youngest children within the same school grade may be more likely to be prescribed speech therapy. To our knowledge, no other study has investigated this association to date. However, studies have examined the relative age effect between children in the same age group for learning difficulties or academic achievement. They showed a 30% to 100% higher risk of diagnoses related to learning difficulties^20,22^ or lower scholastic results among younger vs older children,^20,22,32,33^ consistent with our findings for speech therapy initiation, which is mainly indicated for specific language and learning disorders in children aged 5 to 10 years. We observed a relative age effect associated with speech therapy use in all subgroup analyses according to sex, birth rank among siblings, gestational age, and socioeconomic level. The relative age effect observed for initiation of speech therapy was of the same order of magnitude as that found for initiation of methylphenidate to treat ADHD in both our study and other international studies.^16,20,34,35^

It is unlikely that our findings are associated with a biological mechanism linking the season of birth and incidence of specific language and learning disorders and their management. In our study, the incidence rates of speech therapy use fell sharply between children born in December and those born in January of the following year and attending the next lower grade. Environmental risk factors at birth are unlikely to change abruptly between 2 consecutive months in the winter season. Moreover, previous studies have shown that school entry deadlines, even in different seasons, delimited significant changes in rates of diagnosis or treatment of ADHD.^16,20,32,33^

We expected an influence of the school environment, particularly in the absence of a relative age effect on desmopressin initiation (negative control outcome) in our study. There is growing evidence that children’s relative age, as an indicator of their cognitive and psychoaffective development, may be associated with their performance and behavior at school.^22,36,37^ Two hypotheses may explain our results. First, children born within a few months of the school entry deadline, generally the youngest and least mature in their class, might be subjected to overly high demands, especially in the early school years. These children may be less attentive, more hyperactive, and exhibit weaker language development and poorer academic performance than their older classmates and, consequently, may be more likely to be referred for specialized assessments.^38^ Thus, if tested more frequently, younger children in a class might receive more diagnoses of neurodevelopmental disorders, such as learning disabilities or ADHD, and care.^20,22^ A lack of a relative age effect or an attenuated effect was reported for the diagnosis or treatment of ADHD in countries or districts where school entry is commonly delayed depending on the child’s maturity (ie, Denmark,^39,40^ Israel,^41^ Scotland,^42^ and New South Wales^43^ vs other Australian states with low rates of delayed school entry^44^), consistent with this first hypothesis.

A second, opposite interpretation of the influence of relative age within the class is also possible. Children born earlier and who are, on average, more mature, may find it easier to adapt and, therefore, compensate for clinically relevant symptoms associated with certain neurodevelopmental disorders (ie, learning disabilities, ADHD), which may lead to a reduced likelihood of receiving a diagnosis or treatment.^20^ This possibility is particularly important to consider in the context of limited health care delivery in France for speech therapy in certain parts of the country and child psychiatry throughout the country. Moreover, a recent meta-analysis showed that a diagnosis of ADHD in the youngest children in a class was no more likely to be overturned over time than that of older children in the class, supporting this second hypothesis.^45^

The risk of initiating speech therapy was always higher among the youngest children, regardless of their grade, and the association predominated for pupils in kindergarten and 1st grade and then diminished. In most other studies that have investigated the relative age effect on the diagnosis or management of ADHD, the influence was more pronounced in grades that included children younger than 10 years^16^ and may be explained by the age difference being greater in relative terms among younger children.

The lack of change in the relative age effect observed for speech therapy initiation in the analyses according to the calendar year of inclusion of the children may indicate a lack of knowledge in educational and health care communities of this effect in France. The relative age effect was greater among children who had undergone a speech therapy session after the initial checkup than among those who had only received an initial checkup, whereas the checkup should normally have led to a reduction in the relative age effect in favor of considering the children’s actual age.

### Limitations

This study had some limitations. First, it was not completely exhaustive due to the exclusion of children lacking a linking identifier to their mother’s data (eg, children born abroad or adopted) and the exclusion of same-sex twins for whom certain characteristics cannot be differentiated. However, this limitation concerned only a minority of children. Second, it is possible that some children were wrongly considered within their theoretical grade when they were enrolled in higher grades (eg, in cases of early school entry, particularly for children born in January and February, or in cases of skipping classes), which would mainly contribute to reducing the relative age effect when compared with children enrolled in their theoretical grade. However, such situations are exceptional in France, especially in kindergarten and elementary school. In addition, early transfers of children born at the beginning of the year would not alter the results of analyses evaluating the relative age effect by comparing children born at the end of the year with children born in the second trimester or June.

## Conclusions

In this cohort study in France, the youngest children in a school class were more likely to receive speech therapy compared with the older children. The magnitude of this relative age effect is clinically relevant. Parents, teachers, prescribing physicians, and speech therapists should be aware that age-related relative immaturity may be misdiagnosed as a learning disability or that relative maturity may lead to underdiagnosis of this disorder and adapt their teaching, diagnostic, and therapeutic practices accordingly. Legislators and school administrators might consider introducing greater flexibility for school entry depending on the child’s maturity, month of birth, and possible prematurity.

## Notes

Supplement 1. **eTable 1.** List of Exclusion Diagnoses and Prescriptions **eTable 2.** Organization of the School System in France for Children Aged 3 to 10 Years (Primary School) **eTable 3.** Adjusted Hazard Ratios for the Association Between the Birth Semester and the Initiation of Speech Therapy, Subgroup Analyses **eTable 4.** Adjusted Hazard Ratios for the Association Between the Quarter of Birth and the Initiation of Speech Therapy, Variation in Length of Follow-Up **eTable 5.** Adjusted Hazard Ratios for the Association Between the Quarter of Birth and the Initiation of Speech Therapy, by Year of Inclusion in the Study **eTable 6.** Adjusted Hazard Ratios for the Association Between the Quarter of Birth and the First Checkup for Speech Therapy Followed or Not by Rehabilitation **eTable 7.** Characteristics of Children Initiating Methylphenidate (Positive Control Outcome) or Desmopressin (Negative Control Outcome) Between the Years of the 5th and 10th Birthdays **eTable 8.** Adjusted Hazard Ratios for the Association Between the Quarter of Birth and the Initiation of Desmopressin **eFigure 1.** Follow-Up Scheme **eFigure 2.** Incidence Rate of Methylphenidate Prescription (Positive Control Outcome) by Birth Month and Calendar Year of Study Entry **eFigure 3.** Adjusted Hazard Ratios for Associations Between the Date of Birth (Quarter or Month) and the Initiation of Methylphenidate Between the Years of the 5th and 10th Birthdays

Supplement 2.
Data Sharing Statement
