# Supplementary material for: Relative Age in School and Initiation of Speech Therapy in Children
Source: JAMA Netw Open. 2025 May 23;8(5):e2512262. doi: 10.1001/jamanetworkopen.2025.12262 (PMC12102708; doi:10.1001/jamanetworkopen.2025.12262)
Supplement: Supplement 1. — eTable 1. List of Exclusion Diagnoses and Prescriptions eTable 2. Organization of the School System in France for Children Aged 3 to 10 Years (Primary School) eTable 3. Adjusted Hazard Ratios for the Association Between the Birth Semester and the Initiation of Speech Therapy, Subgroup Analyses eTable 4. Adjusted Hazard Ratios for the Association Between the Quarter of Birth and the Initiation of Speech Therapy, Variation in Length of Follow-Up eTable 5. Adjusted Hazard Ratios for the Association Between the Quarter of Birth and the Initiation of Speech Therapy, by Year of Inclusion in the Study eTable 6. Adjusted Hazard Ratios for the Association Between the Quarter of Birth and the First Checkup for Speech Therapy Followed or Not by Rehabilitation eTable 7. Characteristics of Children Initiating Methylphenidate (Positive Control Outcome) or Desmopressin (Negative Control Outcome) Between the Years of the 5th and 10th Birthdays eTable 8. Adjusted Hazard Ratios for the Association Between the Quarter of Birth and the Initiation of Desmopressin eFigure 1. Follow-Up Scheme eFigure 2. Incidence Rate of Methylphenidate Prescription (Positive Control Outcome) by Birth Month and Calendar Year of Study Entry eFigure 3. Adjusted Hazard Ratios for Associations Between the Date of Birth (Quarter or Month) and the Initiation of Methylphenidate Between the Years of the 5th and 10th Birthdays [file jamanetwopen-e2512262-s001.pdf]

Supplemental Online Content

Billioti de Gage S, Peyre H, Chalumeau M, Mikaeloff Y, Zureik M, Weill A. Relative age in school and initiation of speech therapy in children. *JAMA Netw Open*. 2025;8(5):e2512262. doi:10.1001/jamanetworkopen.2025.12262

eTable 1. List of Exclusion Diagnoses and Prescriptions

eTable 2. Organization of the School System in France for Children Aged 3 to 10 Years (Primary School)

eTable 3. Adjusted Hazard Ratios for the Association Between the Birth Semester and the Initiation of Speech Therapy, Subgroup Analyses

eTable 4. Adjusted Hazard Ratios for the Association Between the Quarter of Birth and the Initiation of Speech Therapy, Variation in Length of Follow-Up

eTable 5. Adjusted Hazard Ratios for the Association Between the Quarter of Birth and the Initiation of Speech Therapy, by Year of Inclusion in the Study

eTable 6. Adjusted Hazard Ratios for the Association Between the Quarter of Birth and the First Checkup for Speech Therapy Followed or Not by Rehabilitation

eTable 7. Characteristics of Children Initiating Methylphenidate (Positive Control Outcome) or Desmopressin (Negative Control Outcome) Between the Years of the 5th and 10th Birthdays

eTable 8. Adjusted Hazard Ratios for the Association Between the Quarter of Birth and the Initiation of Desmopressin

eFigure 1. Follow-Up Scheme

eFigure 2. Incidence Rate of Methylphenidate Prescription (Positive Control Outcome) by Birth Month and Calendar Year of Study Entry

eFigure 3. Adjusted Hazard Ratios for Associations Between the Date of Birth (Quarter or Month) and the Initiation of Methylphenidate Between the Years of the 5th and 10th Birthdays

This supplemental material has been provided by the authors to give readers additional information about their work.

© 2025 Billioti de Gage S et al. *JAMA Network Open*.

eTable 1. List of Exclusion Diagnoses and Prescriptions

ICD 10 code for excluded diagnoses	
Congenital malformations, deformations and chromosomal abnormalities	
Q00	Anencephaly and similar malformations
Q01	Encephalocele
Q02	Microcephaly
Q03	Congenital hydrocephalus
Q04	Other congenital malformations of brain
Q05.0 to Q05.4	Spina bifida with hydrocephalus
Q90	Down syndrome
Mental and behavioural disorders; Alzheimer's disease	
Organic, including symptomatic, mental disorders; Alzheimer's disease	
F00 to F09	Organic, including symptomatic, mental disorders
F99	Unspecified mental disorders
G30	Alzheimer disease
Schizophrenia, schizotypal and delusional disorders:	
F20	Schizophrenia
F21	Schizotypal disorders
F22	Persistent delusional disorders
F23	Acute and transient psychotic disorders
F24	Induced delusional disorder
F25	Schizoaffective disorders
F28	Other nonorganic psychotic disorders
F29	Unspecified nonorganic psychosis
Mood [affective] disorders:	
F30	Manic episode
F31	Bipolar affective disorder
F32	Depressive episode
F33	Recurrent depressive disorder
F34	Persistent mood [affective] disorders
F38	Other mood [affective] disorders
F39	Unspecified mood [affective] disorder
Neurotic, stress-related and somatoform disorders:	
F40	Phobic anxiety disorders
F41	Other anxiety disorders
F42	Obsessive-compulsive disorder
F43	Reaction to severe stress, and adjustment disorders
F44	Dissociative [conversion] disorders
F45	Somatoform disorders
F48	Other neurotic disorders
Mental retardation:	
F70	Mild mental retardation
F71	Moderate mental retardation
F72	Severe mental retardation
F73	Profound mental retardation
F78	Other mental retardation
F79	Unspecified mental retardation
Disorders of psychological development:	
F80	Specific developmental disorders of speech and language
F81	Specific developmental disorders of scholastic skills
F82	Specific developmental disorder of motor function
F83	Mixed specific developmental disorders
F84	Pervasive developmental disorders
F88	Other disorders of psychological development
F89	Unspecified disorder of psychological development
Behavioural and emotional disorders with onset usually occurring in childhood and adolescence; other	
F90	Hyperkinetic disorders
F91	Conduct disorders
F92	Mixed disorders of conduct and emotions
F93	Emotional disorders with onset specific to childhood
F94	Disorders of social functioning with onset specific to childhood and adolescence
F95	Tic disorders
F98	Other behavioural and emotional disorders with onset usually occurring in childhood and adolescence
F50-63	Other
Diseases of the nervous system	
G47.4	Narcolepsy and cataplexy
G40-41	Epilepsy, status epilepticus

© 2025 Billioti de Gage S et al. *JAMA Network Open*.

eTable 1. List of Exclusion Diagnoses and Prescriptions *(continued)*

ATC code for excluded prescriptions[a]	
Antipsychotics	
N05AA	Phenothiazines with aliphatic side-chain
N05AB	Phenothiazines with piperazine structure
N05AC	Phenothiazines with piperidine structure
N05AD	Butyrophenone derivatives
N05AF	Thioxanthene derivatives
N05AG	Diphenylbutylpiperidine derivatives
N05AH	Diazepines, oxazepines, thiazepines and oxepines
N05AL	Benzamides
N05AX	Other antipsychotics
Anxiolytics	
N05BA	Benzodiazepine derivatives
N05BB01	Diphenylmethane derivatives
N05BC01	Meprobamate
N05BE01	Buspirone
N05BX03	Etifoxine
Hypnotics and sedatives	
N05CD	Benzodiazepine derivatives
N05CF	Benzodiazepine related drugs
N05CX	Hypnotics and sedatives in combination, excluding barbiturates
N05CH01	Melatonin
R06AD01	Alimemazine
Antidepressants	
N06AA	Non-selective monoamine reuptake inhibitors
N06AB	Selective serotonin reuptake inhibitors
N06AF	Monoamine oxidase inhibitors, non-selective
N06AG	Monoamine oxidase A inhibitors
N06AX	Other antidepressants
Mood regulators	
N05AN01	Lithium
N03AG01	Sodium divalproate
N03AG02	Valpromide
Psychostimulants	
N06BA04	Methylphenidate

[a]*Exclusion if at least two prescriptions on different dates in the two years preceding inclusion from the same therapeutic family.*

© 2025 Billioti de Gage S et al. *JAMA Network Open*.

eTable 2. Organization of the School System in France for Children Aged 3 to 10 Years (Primary School)

School entry age: September of the year in which the children reach the age of 3

Period considered for entering a grade: January 1 to December 31

Period of the school year: September to the next June or July

Age reached during the period considered for entering a grade	3 years	4 years	5 years	6 years	7 years	8 years	9 years	10 years
Cycle	Nursery school (Kindergarten)			Elementary school				
Grade	1st [petite section]	2nd [moyenne section]	3rd [grande section]	1st [CP]	2nd [CE1]	3rd [CE2]	4th [CM1]	5th [CM2]
Main learning	Preparation for basic learning			Fundamental learning (reading, writing, arithmetic, respect for others)			Stabilization and consolidation of basic learning	

© 2025 Billioti de Gage S et al. *JAMA Network Open*.

eTable 3. Adjusted Hazard Ratios for the Association Between the Birth Semester and the Initiation of Speech Therapy, Subgroup Analyses

Subgroup<br>Semester of birth	Speech therapy<br>treated<br>n=692 086	 not treated<br>n=3 496 899	 adjusted HR<br>(95% CI)[a]
SEX			p=0.01
**Boys:**	**n=377 273**	**n=1 683 034**	
January-June	162 179	846 017	1.00 (ref.)
July-December	215 094	837 017	1.31 (1.30-1.32)
**Girls:**	**n=314 813**	**n=1 813 865**	
January-June	133 347	905 711	1.00 (ref.)
July-December	181 466	908 154	1.33 (1.32-1.34)
SIBLING RANK			p=0.46
**First:**	**n=401 343**	**n=2 042 807**	
January-June	174 009	1 032 648	1.00 (ref.)
July-December	227 334	1 010 159	1.32 (1.31-1.33)
**Second:**	**n=248 523**	**n=1 198 037**	
January-June	104 940	598 583	1.00 (ref.)
July-December	143 583	599 454	1.33 (1.32-1.34)
**≥Third:**	**n=41 974**	**n=255 042**	
January-June	16 464	119 992	1.00 (ref.)
July-December	25 510	135 050	1.31 (1.28-1.33)
SOLIDARITY-BASED HEALTH INSURANCE			p=0.14
**Affiliated:**	**n=69 362**	**n=371 180**	
January-June	28 769	179 322	1.00 (ref.)
July-December	40 593	191 858	1.31 (1.29-1.33)
**Not affiliated:**	**n=621 649**	**n=3 119 832**	
January-June	266 294	1 569 403	1.00 (ref.)
July-December	355 355	1 550 429	1.32 (1.32-1.33)
DISADVANTAGE INDEX, *quintiles*			p=0.03
**Q1** ***(least disadvantaged)*****:**	**n=138 525**	**n=670 561**	
January-June	59 763	337 609	1.00 (ref.)
July-December	78 762	332 952	1.31 (1.30-1.33)
**Q2 to Q4:**	**n=409 553**	**n=1 958 412**	
January-June	174 810	980 513	1.00 (ref.)
July-December	234 743	977 899	1.32 (1.31-1.33)
**Q5** ***(most disadvantaged)*****:**	**n=115 709**	**n=663 853**	
January-June	48 747	330 374	1.00 (ref.)
July-December	66 962	333 479	1.34 (1.32-1.36)
PREMATURITY			p=0.74
**Premature:**	**n=44 833**	**n=194 788**	
January-June	19 260	98 379	1.00 (ref.)
July-December	25 573	96 409	1.32 (1.30-1.35)
**Not premature:**	**n=647 240**	**n=3 302 031**	
January-June	276 261	1 653 317	1.00 (ref.)
July-December	370 979	1 648 714	1.32 (1.31-1.33)

HR: hazard ratio.

[a]Models adjusted for birth quarter, sex, gestational age, weight for gestational age, birth rank, in-utero exposure to tobacco, alcohol, psychotropic drugs, or valproic acid, solidarity-based complementary health insurance, disadvantage index of municipality of residence, size of urban area of residence, French region of residence, and calendar year of inclusion.

© 2025 Billioti de Gage S et al. *JAMA Network Open*.

eTable 4. Adjusted Hazard Ratios for the Association Between the Quarter of Birth and the Initiation of Speech Therapy, Variation in Length of Follow-Up ***(from September 1 of the fifth birthday year to July 31 of the sixth to 10th birthday years)***

**Age at study end** *(31 July of sixth to 10th birthday year)*	**Speech therapy**		
	**treated**	**not treated**	**adjusted HR (95% CI)[a]**
Sixth birthday year *(end of nursery school)*	n=214 399	n=3 974 586	
**Quarter of birth:**			
January-March	39 498	976 181	1.00 (ref.)
April-June	46 693	984 882	1.17 (1.15-1.18)
July-September	59 538	1 031 163	1.41 (1.39-1.43)
October-December	68 670	982 360	1.70 (1.68-1.72)
Seventh birthday year *(end of first elementary class)*	n=411 458	n=3 777 527	
**Quarter of birth:**			
January-March	78 514	937 165	1.00 (ref.)
April-June	91 384	940 191	1.15 (1.14-1.16)
July-September	113 897	976 804	1.37 (1.36-1.38)
October-December	127 663	923 367	1.61 (1.60-1.63)
Eighth birthday year *(end of second elementary class)*	n=563 961	n=3 625 024	
**Quarter of birth:**			
January-March	110 399	905 280	1.00 (ref.)
April-June	127 192	904 383	1.14 (1.13-1.15)
July-September	154 908	935 793	1.33 (1.32-1.34)
October-December	171 462	879 568	1.55 (1.54-1.57)
Ninth birthday year *(end of third elementary class)*	n=648 331	n=3 540 654	
**Quarter of birth:**			
January-March	128 501	887 178	1.00 (ref.)
April-June	146 994	884 581	1.14 (1.13-1.14)
July-September	177 645	913 056	1.32 (1.31-1.33)
October-December	195 191	855 839	1.53 (1.52-1.54)
10th birthday year[b] *(end of fourth elementary class)*	n=692 086	n=3 496 899	
**Quarter of birth:**			
January-March	138 075	877 604	1.00 (ref.)
April-June	157 451	874 124	1.13 (1.12-1.14)
July-September	189 464	901 237	1.31 (1.30-1.32)
October-December	207 096	843 934	1.51 (1.50-1.52)

HR: hazard ratio.
[a]Models adjusted for birth quarter, sex, gestational age, weight for gestational age, birth rank, in-utero exposure to tobacco, alcohol, psychotropic drugs, or valproic acid, solidarity-based complementary health insurance, disadvantage index of municipality of residence, size of urban area of residence, French region of residence, and calendar year of inclusion.
[b]Main analysis.

© 2025 Billioti de Gage S et al. *JAMA Network Open*.

eTable 5. Adjusted Hazard Ratios for the Association Between the Quarter of Birth and the Initiation of Speech Therapy, by Year of Inclusion in the Study ***(maximum follow-up, 3 years)***

Inclusion year (September 1 of fifth birthday year)	Speech therapy		
	treated	not treated	adjusted HR (95% CI)[a]
2015	n=99 076	n=479 578	
**Quarter of birth:**			
January-March	19 732	120 017	1.00 (ref.)
April-June	22 572	119 783	1.13 (1.11-1.15)
July-September	26 654	122 373	1.29 (1.27-1.32)
October-December	30 118	117 405	1.50 (1.47-1.53)
2016	n=98 997	n=499 823	
**Quarter of birth:**			
January-March	19 442	124 696	1.00 (ref.)
April-June	22 651	126 486	1.13 (1.11-1.16)
July-September	27 361	129 214	1.33 (1.30-1.35)
October-December	29 543	119 427	1.53 (1.50-1.56)
2017	n=93 748	n=516 685	
**Quarter of birth:**			
January-March	18 339	129 574	1.00 (ref.)
April-June	21 302	128 575	1.16 (1.13-1.18)
July-September	25 591	132 608	1.33 (1.31-1.36)
October-December	28 516	125 928	1.55 (1.52-1.58)
2018	n=90 981	n=515 442	
**Quarter of birth:**			
January-March	17 743	128 022	1.00 (ref.)
April-June	20 221	127 537	1.13 (1.11-1.16)
July-September	25 212	134 041	1.33 (1.30-1.36)
October-December	27 805	125 842	1.55 (1.52-1.58)
2019	n=88 732	n=520 829	
**Quarter of birth:**			
January-March	17 713	132 984	1.00 (ref.)
April-June	20 019	129 309	1.15 (1.13-1.17)
July-September	24 352	133 304	1.34 (1.32-1.37)
October-December	26 648	125 232	1.56 (1.53-1.59)
2015 to 2019	n=471 534	n=2 532 357	
**Quarter of birth:**			
January-March	92 969	635 293	1.00 (ref.)
April-June	106 765	631 690	1.14 (1.13-1.15)
July-September	129 170	651 540	1.32 (1.31-1.34)
October-December	142 630	613 834	1.54 (1.52-1.55)

HR: hazard ratio.

[a]Models adjusted for birth quarter, sex, gestational age, weight for gestational age, birth rank, in-utero exposure to tobacco, alcohol, psychotropic drugs, or valproic acid, solidarity-based complementary health insurance, disadvantage index of municipality of residence, size of urban area of residence, region of residence, and calendar year of inclusion.

© 2025 Billioti de Gage S et al. *JAMA Network Open*.

eTable 6. Adjusted Hazard Ratios for the Association Between the Quarter of Birth and the First Checkup for Speech Therapy Followed or Not by Rehabilitation

Speech therapy	treated	not treated	adjusted HR (95% CI)[a]
Check-up without rehabilitation	n=136 254	n=3 496 899	
**Quarter of birth:**			
January-March	27 954	877 604	1.00 (ref.)
April-June	31 381	874 124	1.13 (1.11-1.14)
July-September	37 192	901 237	1.30 (1.28-1.32)
October-December	39 727	843 934	1.48 (1.46-1.50)
Check-up with rehabilitation	n=555 166	n=3 496 899	
**Quarter of birth:**			
January-March	109 984	877 604	1.00 (ref.)
April-June	125 917	874 124	1.14 (1.13-1.15)
July-September	152 097	901 237	1.33 (1.32-1.34)
October-December	167 168	843 934	1.54 (1.53-1.55)

HR: hazard ratio.

[a]Models adjusted for birth quarter, sex, gestational age, weight for gestational age, birth rank, in-utero exposure to tobacco, alcohol, psychotropic drugs, or valproic acid, solidarity-based complementary health insurance, disadvantage index of municipality of residence, size of urban area of residence, French region of residence, and calendar year of inclusion.

© 2025 Billioti de Gage S et al. *JAMA Network Open*.

eTable 7. Characteristics of Children Initiating Methylphenidate (Positive Control Outcome) or Desmopressin (Negative Control Outcome) Between the Years of the 5th and 10th Birthdays

	Methylphenidate		Desmopressin	
	treated N=38 794 (%)	not treated N=4 731 043 (%)	treated N=58 589 (%)	not treated N=4 708 638 (%)
**Sex:**				
Male	30 347 (78.2)	2 398 703 (50.7)	38 856 (66.3)	2 388 784 (50.7)
Female	8447 (21.8)	2 332 340 (49.3)	19 733 (33.7)	2 319 854 (49.3)
**Gestational age,** weeks:				
Non missing	*38 790 (100.0)*	*4 730 925 (100.0)*	*58 588 (100.0)*	*4 708 517 (100.0)*
22-27 *(extremely preterm)*	200 (0.5)	7286 (0.2)	125 (0.2)	7358 (0.2)
28-31 *(very preterm)*	429 (1.1)	24 438 (0.5)	379 (0.7)	24 476 (0.5)
32-36 *(moderate to late preterm)*	2740 (7.1)	246 851 (5.2)	3241 (5.5)	246 196 (5.2)
37-40 *(full-term)*	28 784 (74.2)	3 599 422 (76.1)	44 452 (75.9)	3 581 740 (76.1)
>40 *(term exceeded or post-term)*	6637 (17.1)	852 928 (18.0)	10 391 (17.7)	848 747 (18.0)
**Weight for gestational age,** percentile[a]:				
Non missing	*37 228 (96.0)*	*4 565 996 (96.5)*	*56 257 (96.0)*	*4 544 459 (96.5)*
<3rd^{ème} *(severe low weight)*	2347 (6.1)	184 284 (3.9)	2116 (3.6)	184 433 (3.9)
<10th^{ème} *(small for gestational age)*	3421 (8.8)	349 308 (7.4)	4088 (7.0)	348 460 (7.4)
10-90th^{ème} *(normal weight)*	27 562 (71.1)	3 517 070 (74.3)	42 754 (73.0)	3 499 956 (74.3)
>90th^{ème} *(macrosomia)*	2294 (5.9)	306 952 (6.5)	4228 (7.2)	304 833 (6.5)
>97th^{ème} *(severe macrosomia)*	1604 (4.1)	208 382 (4.4)	3071 (5.2)	206 777 (4.4)
**In utero exposure,** *vs.* non exposure:				
**Tobacco**	4720 (12.2)	346 415 (7.3)	4244 (7.2)	346 693 (7.4)
**Alcohol**	524 (1.4)	26 973 (0.6)	355 (0.6)	27 124 (0.6)
**Psychotropic drugs**	2964 (7.6)	187 255 (4.0)	2955 (5.0)	187 126 (4.0)
**Valproic acid**	84 (0.2)	4253 (0.1)	76 (0.1)	4255 (0.1)
**Birth rank** *(since 2006)*:				
Non missing	*38 770 (99.9)*	*4 729 529 (100.0)*	*58 568 (100.0)*	*4 707 123 (100.0)*
1	24 530 (63.2)	2 750 283 (58.1)	34 256 (58.5)	2 739 089 (58.2)
2	12 155 (31.4)	1 644 903 (34.8)	20 826 (35.6)	1 635 262 (34.7)
≥3	2085 (5.4)	334 343 (7.1)	3486 (6.0)	332 772 (7.1)
**Solidarity-based complementary health insurance:**				
Non missing	*38 728 (99.8)*	*4 723 246 (99.8)*	*58 519 (99.9)*	*4 700 853 (99.8)*
No	34 900 (90.0)	4 225 907 (89.3)	51 867 (88.5)	4 206 655 (89.3)
Yes	3828 (9.9)	497 339 (10.5)	6652 (11.4)	494 198 (10.5)
**Disadvantage index[b],** quintile:				
Non missing	*37 575 (96.9)*	*4 472 648 (94.5)*	*56 733 (96.8)*	*4 451 008 (94.5)*
Q1 *(less disadvantaged)*	7637 (19.7)	907 086 (19.2)	9564 (16.3)	904 699 (19.2)
Q2	7570 (19.5)	923 262 (19.5)	11 250 (19.2)	919 113 (19.5)
Q3	7487 (19.3)	888 389 (18.8)	11 295 (19.3)	884 104 (18.8)
Q4	7472 (19.3)	872 041 (18.4)	11 537 (19.7)	867 474 (18.4)
Q5 *(more disadvantaged)*	7409 (19.1)	881 870 (18.6)	13 087 (22.3)	875 618 (18.6)
**Size of urban area of residence:**				
Non missing	*37 945 (97.8)*	*4 629 220 (97.8)*	*57 494 (98.1)*	*4 607 107 (97.8)*
Rural area	8651 (22.3)	960 652 (20.3)	11 858 (20.2)	956 917 (20.3)
2000 to 9999 inhabitants	5523 (14.2)	599 235 (12.7)	7335 (12.5)	597 127 (12.7)
10 000 to 49 999 inhabitants	4417 (11.4)	504 347 (10.7)	6056 (10.3)	502 459 (10.7)
50 000 to 199 999 inhabitants	4888 (12.6)	600 699 (12.7)	7538 (12.9)	597 689 (12.7)
≥200 000 inhabitants	14 466 (37.3)	1 964 287 (41.5)	24 707 (42.2)	1 952 915 (41.5)
**French region of residence:**				
Non missing	*38 793 (100.0)*	*4 731 002 (100.0)*	*58 588 (100.0)*	*4 708 597 (100.0)*
Auvergne-Rhône-Alpes	4990 (12.9)	574 415 (12.1)	6017 (10.3)	573 114 (12.2)
Bourgogne-Franche-Comté	1243 (3.2)	179 092 (3.8)	2291 (3.9)	177 962 (3.8)
Bretagne	1624 (4.2)	233 472 (4.9)	2955 (5.0)	232 052 (4.9)
Centre-Val de Loire	1356 (3.5)	181 197 (3.8)	2440 (4.2)	180 025 (3.8)
Corse	112 (0.3)	16 014 (0.3)	114 (0.2)	16 003 (0.3)
Grand Est	3670 (9.5)	355 685 (7.5)	5211 (8.9)	353 930 (7.5)
Hauts-de-France	4289 (11.1)	452 835 (9.6)	7505 (12.8)	449 318 (9.5)
Ile-de-France	6045 (15.6)	907 259 (19.2)	9668 (16.5)	903 178 (19.2)
Normandie	2603 (6.7)	231 205 (4.9)	3444 (5.9)	230 217 (4.9)
Nouvelle-Aquitaine	2996 (7.7)	388 757 (8.2)	4697 (8.0)	386 865 (8.2)
Occitanie	4784 (12.3)	396 260 (8.4)	5318 (9.1)	395 516 (8.4)
Pays de la Loire	1554 (4.0)	285 967 (6.0)	3541 (6.0)	283 760 (6.0)
Provence-Alpes-Côte d'Azur	3029 (7.8)	353 380 (7.5)	4459 (7.6)	351 716 (7.5)
French overseas region	498 (1.3)	175 464 (3.7)	928 (1.6)	174 941 (3.7)

© 2025 Billioti de Gage S et al. *JAMA Network Open*.

eTable 7. Characteristics of Children Initiating Methylphenidate (Positive Control Outcome) or Desmopressin (Negative Control Outcome) Between the Years of the 5th and 10th Birthdays *(continued)*

	Methylphenidate		Desmopressin	
	treated N=38 794 (%)	not treated N=4 731 043 (%)	treated N=58 589 (%)	not treated N=4 708 638 (%)
**Year of fifth birthday[c]:**				
2015	7043 (18.2)	651 417 (13.8)	12 635 (21.6)	645 333 (13.7)
2016	7920 (20.4)	674 789 (14.3)	12 247 (20.9)	669 997 (14.2)
2017	8577 (22.1)	688 076 (14.5)	11 904 (20.3)	684 342 (14.5)
2018	6960 (17.9)	685 443 (14.5)	9512 (16.2)	682 536 (14.5)
2019	5266 (13.6)	691 408 (14.6)	7113 (12.1)	689 198 (14.6)
2020	2551 (6.6)	679 088 (14.4)	4137 (7.1)	677 230 (14.4)
2021	477 (1.2)	660 822 (14.0)	1041 (1.8)	660 002 (14.0)
**Quarter of birth:**				
January-March	7704 (19.9)	1 142 774 (24.2)	14 757 (25.2)	1 134 764 (24.1)
April-June	8544 (22.0)	1 160 984 (24.5)	14 321 (24.4)	1 154 503 (24.5)
July-September	10 808 (27.9)	1 232 508 (26.1)	15 256 (26.0)	1 227 509 (26.1)
October-December	11 738 (30.3)	1 194 777 (25.3)	14 255 (24.3)	1 191 862 (25.3)
**Month of birth:**				
January	2557 (6.6)	395 989 (8.4)	5196 (8.9)	392 984 (8.4)
February	2504 (6.5)	361 760 (7.7)	4611 (7.9)	359 357 (7.6)
March	2643 (6.8)	385 025 (8.1)	4950 (8.5)	382 423 (8.1)
April	2618 (6.8)	372 392 (7.9)	4636 (7.9)	370 125 (7.9)
May	2920 (7.5)	397 127 (8.4)	4923 (8.4)	394 874 (8.4)
June	3006 (7.8)	391 465 (8.3)	4762 (8.1)	389 504 (8.3)
July	3482 (9.0)	416 837 (8.8)	5150 (8.8)	414 960 (8.8)
August	3667 (9.5)	411 153 (8.7)	5125 (8.8)	409 516 (8.7)
September	3659 (9.4)	404 518 (8.6)	4981 (8.5)	403 033 (8.6)
October	3890 (10.0)	411 528 (8.7)	4939 (8.4)	410 332 (8.7)
November	3858 (9.9)	387 100 (8.2)	4572 (7.8)	386 263 (8.2)
December	3990 (10.3)	396 149 (8.4)	4744 (8.1)	395 267 (8.4)

[a]*Adjusted for gestational age and sex, Z-score.*
[b]*Disadvantage index of the municipality of residence.*
[c]*Due to the study design, the maximum duration of follow-up was shorter for children included after 2017.*

Compared to children not treated with methylphenidate between the ages of five and 10, treated children were more likely to be boys (78.2% vs. 50.7%), to be born prematurely (8.7% vs. 5.9%), to be born with low weight for gestational age (14.9% vs. 11.3%), to be exposed in utero to tobacco (12.2% vs. 7.3%), alcohol (1.4% vs. 0.6%), psychotropic drugs (7.6% vs. 4.0%), or valproic acid (0.20% vs. 0.09%), to be the first sibling (63.2% vs. 58.1%), to live in more privileged municipalities (39.2% vs. 38.7% for the first two quintiles of the disadvantage index) or in an urban area of less than 200 000 inhabitants (60.5% vs. 56.4%), to be born at the end of the year (30.3% vs. 25.3% born in the last quarter) and less likely to be affiliated to the solidarity-based complementary health insurance (9.9% vs. 10.5%). Compared with children without methylphenidate treatment, those treated were more likely to live in the Occitanie (12.3% vs. 8.4%) or Grand Est (9.5% vs. 7.5%) regions, and less likely to live in the Pays de la Loire (4.0% vs. 6.0%), Ile-de-France [including Paris] (15.6% vs. 19.2%), or French overseas (1.3% vs. 3.7%) regions.

Compared to children not treated with desmopressin between the ages of five and 10, treated children were more likely to be boys (66.3% vs. 50.7%), to be born prematurely (6.4% vs. 5.9%), to be exposed in utero to psychotropic drugs (5.0% vs. 4.0%), to be the first or second sibling (94.1 vs. 92.9), to be affiliated to the solidarity-based complementary health insurance (11.4% vs. 10.5%), to live in less privileged municipalities (42.0% vs. 37.0% for the last two quintiles of the disadvantage index) or in an urban area of 200 000 inhabitants or more (42.2% vs. 41.5%), to be born at the beginning of the year (25.2% vs. 24.1% born in the first quarter) and less likely to be born with low weight for gestational age (10.6% vs. 11.3%). There was no difference between children treated and not treated with desmopressin in terms of in utero exposure to tobacco (7.2% vs. 7.4%), alcohol (0.6% vs. 0.6%), or valproic acid (0.1% vs. 0.1%). Compared with children without desmopressin treatment, those treated were more likely to live in the Hauts-de-France (12.8% vs. 9.5%) or Grand Est (8.9% vs. 7.5%) regions, and less likely to live in the Ile-de-France [including Paris] (16.5% vs. 19.2%), Auvergne-Rhône-Alpes (10.3% vs. 12.2%), or French overseas (1.6% vs. 3.7%) regions.

eTable 8. Adjusted Hazard Ratios for the Association Between the Quarter of Birth and the Initiation of Desmopressin *(negative control outcome)*

Desmopressin	treated N=58 589	not treated N=4 708 638	adjusted HR (95% CI)[a]
**Quarter of birth:**			
January-March	14 757	1 134 764	1.00 (ref.)
April-June	14 321	1 154 503	0.95 (0.93-0.97)
July-September	15 256	1 227 509	0.95 (0.93-0.97)
October-December	14 255	1 191 862	0.91 (0.89-0.93)

HR: hazard ratio.
[a]*Models adjusted for birth quarter, sex, gestational age, weight for gestational age, birth rank, in-utero exposure to tobacco, alcohol, psychotropic drugs, or valproic acid, solidarity-based complementary health insurance, disadvantage index of municipality of residence, size of urban area of residence, French region of residence, and calendar year of inclusion.*

© 2025 Billioti de Gage S et al. *JAMA Network Open*.

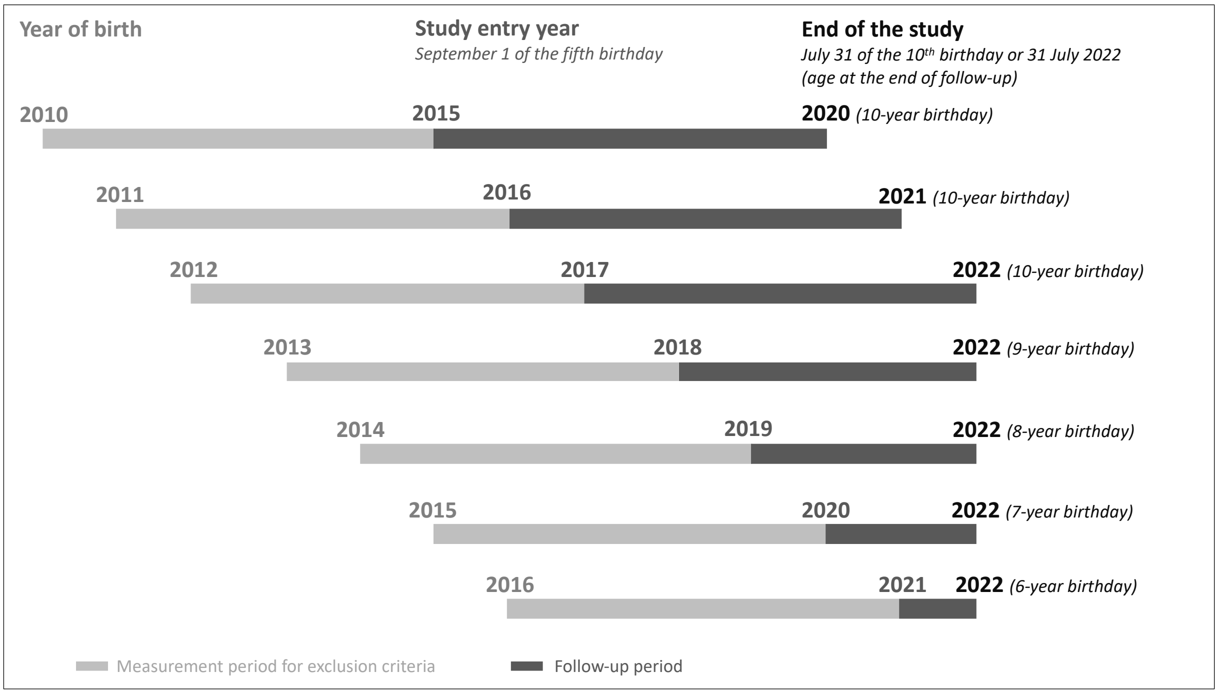

eFigure 1. Follow-Up Scheme

© 2025 Billioti de Gage S et al. *JAMA Network Open*.

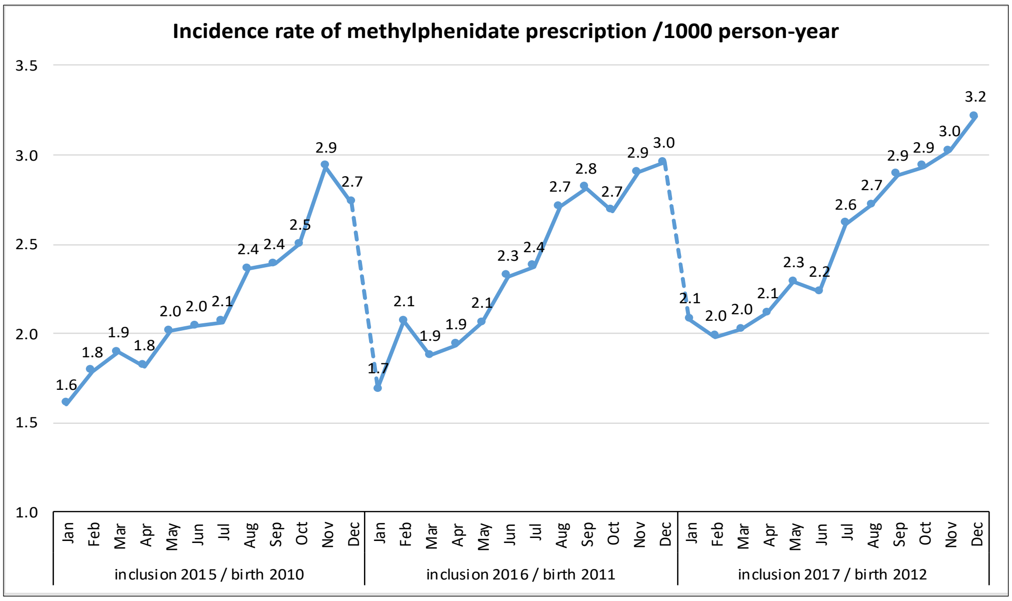

eFigure 2. Incidence Rate of Methylphenidate Prescription (Positive Control Outcome) by Birth Month and Calendar Year of Study Entry ***(for children born between 2010 and 2012, who benefited from the maximum follow-up)***

© 2025 Billioti de Gage S et al. *JAMA Network Open*.

Methylphenidate	treated N=38 794 (%)	not treated N=4 731 043 (%)	adjusted HR1 (95% CI)[a]	adjusted HR2 (95%CI)[a]
**Quarter of birth:**				
January-March	7704 (19.9)	1 142 774 (24.2)	1.00 (ref.)	0.91 (0.89-0.94)
April-June	8544 (22.0)	1 160 984 (24.5)	1.09 (1.06-1.13)	1.00 (ref.)
July-September	10 808 (27.9)	1 232 508 (26.1)	1.30 (1.26-1.34)	1.19 (1.16-1.22)
October-December	11 738 (30.3)	1 194 777 (25.3)	1.45 (1.41-1.49)	1.32 (1.29-1.36)
**Month of birth:**				
January	2557 (6.6)	395 989 (8.4)	1.00 (ref.)	0.84 (0.79-0.88)
February	2504 (6.5)	361 760 (7.7)	1.07 (1.02-1.14)	0.90 (0.85-0.95)
March	2643 (6.8)	385 025 (8.1)	1.06 (1.00-1.12)	0.88 (0.84-0.93)
April	2618 (6.8)	372 392 (7.9)	1.09 (1.03-1.15)	0.91 (0.86-0.96)
May	2920 (7.5)	397 127 (8.4)	1.14 (1.08-1.20)	0.95 (0.91-1.00)
June	3006 (7.8)	391 465 (8.3)	1.20 (1.13-1.26)	1.00 (ref.)
July	3482 (9.0)	416 837 (8.8)	1.29 (1.23-1.36)	1.08 (1.03-1.14)
August	3667 (9.5)	411 153 (8.7)	1.38 (1.31-1.45)	1.15 (1.10-1.21)
September	3659 (9.4)	404 518 (8.6)	1.40 (1.33-1.47)	1.17 (1.11-1.23)
October	3890 (10.0)	411 528 (8.7)	1.46 (1.39-1.53)	1.22 (1.16-1.28)
November	3858 (9.9)	387 100 (8.2)	1.53 (1.46-1.61)	1.28 (1.22-1.34)
December	3990 (10.3)	396 149 (8.4)	1.55 (1.47-1.62)	1.29 (1.23-1.36)

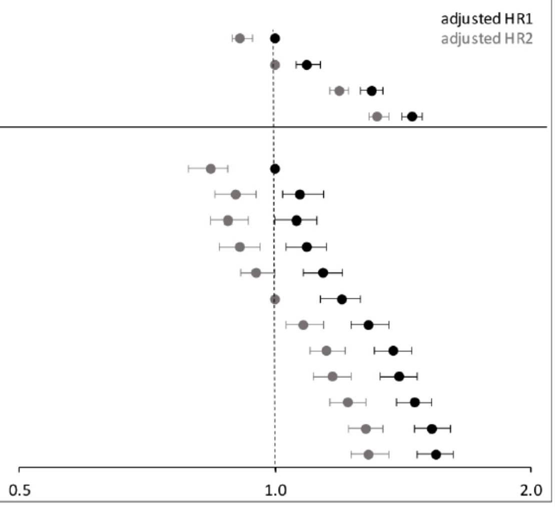

HR: hazard ratio.
[a]Models adjusted for birth quarter (January-March as ref. for HR1 and April-June as ref. for HR2) or month (January as ref. for HR1 and June as ref. for HR2), sex, gestational age, weight for gestational age, birth rank, in utero exposure to tobacco, alcohol, psychotropic drugs, or valproic acid, solidarity-based complementary health insurance, disadvantage index, size of urban area of residence, French region of residence, and calendar year of inclusion.

eFigure 3. Adjusted Hazard Ratios for Associations Between the Date of Birth (Quarter or Month) and the Initiation of Methylphenidate Between the Years of the 5th and 10th Birthdays

© 2025 Billioti de Gage S et al. *JAMA Network Open*.
