# Supplementary material for: Relative Age in School and Initiation of Speech Therapy in Children
Source: JAMA Netw Open. 2025 May 23;8(5):e2512262. doi: 10.1001/jamanetworkopen.2025.12262 (PMC12102708; doi:10.1001/jamanetworkopen.2025.12262)
Supplement: Supplement 2. — Data Sharing Statement [file jamanetwopen-e2512262-s002.pdf]

Data Sharing Statement

Billioti de Gage. Relative Age in School and Initiation of Speech Therapy in Children. *JAMA Netw Open*. Published May 23, 2025. doi:10.1001/jamanetworkopen.2025.12262

Data
Data available: No

Additional Information
Explanation for why data not available: Use of SNDS data is restricted to authorised persons
